# The Concept of Thinking: A Reappraisal of Ryle’s Work

**DOI:** 10.4103/0973-1229.77442

**Published:** 2011

**Authors:** Nilanjan Das

**Affiliations:** * *Graduate Student, Department of Philosophy, Jadavpur University, West Bengal, India.*

**Keywords:** *Thinking*, *Thought*, *Intention-parasitism*, *Narrative*, *Privacy*

## Abstract

In The Concept of Mind, Ryle’s official position seems to be that mental acts cannot be intrinsically private. In The Concept of Mind as well as his later work on thinking, Ryle views thinking as an activity that terminates in a thought, which is a state of being prepared for a performance. Thinking is characterised by what Ryle calls intention-parasitism; for it is, insofar as its underlying motive is concerned, parasitic on the final performance which will take place later. Ryle shows that acts of thinking, owing to their intention-parasitism, can be described in a tactical idiom, with reference to the final performance for which it was intended. However, this framework of intention-parasitism is not adequate to describe all instances of thinking in all their aspects, which therefore remain inextricably private. The task of this paper is to accommodate such privacy within the theoretical framework suggested in The Concept of Mind.

## Introduction

In this paper, I argue that Gilbert Ryle’s attack on “privacy” of mental acts in *The Concept of Mind* (henceforth *CM*) has been misconstrued. The misconstrual consists in identifying Ryle’s concept of mind as a “psychological” one, which interprets *all* mental concepts exhaustively in terms of publicly accessible behaviour or behavioural dispositions. Chalmers (1996, p14) puts forward this characterization, akin to the label of “logical behaviourism” ordinarily ascribed to Ryle. One must note that his theory cannot be psychological in the sense that it is a product of empirical enquiry. It can be psychological in the sense that it interprets mental concepts with reference to observable phenomena and, therefore, provides a conceptual framework which can support psychological research. Indeed, for Ryle (1971, p300), this is one goal of ordinary language philosophy. The reason for this is that according to Ryle, apparently, no mental concept stands for anything that is essentially private and that stands beyond publicly accessible behaviour or behavioural dispositions.

Recent writers such as Byrne (1997) have attempted to adopt a more balanced view regarding Ryle’s work, distinguishing his brand of behaviourism or “quasi-behaviourism” from that of Hempel (1949) who subscribed to a form of physicalism insofar as he considered all statements about mental states translatable into statements about physical phenomena. By contrast, Ryle spoke of agential behavioural dispositions which are different from plain observable physical behaviour. Correspondingly, Woodruff Smith and Thomasson (2005) note Husserl’s non-introspectionist and non-physicalist concern with the essences of mental concepts as a major influence on Ryle’s methodological adherence to conceptual analysis. Yet, in current philosophical literature, including Byrne (1997), Woodruff Smith and Thomasson (2005), Soames (2005) and Anbari (2009), Ryle is still regarded as a staunch opponent of mental privacy in every form.

Here, by examining Ryle’s insights on the concept of “thinking” in *CM* as well as his later work, I shall show that Ryle’s interpretation of mental concepts should not be understood in this manner.

## Thinking of Thoughts

Let us begin with the general distinction drawn in *CM*, between *task verbs* and *achievement verbs*. A task verb denotes an occurrence: an action, an exertion or a performance. An achievement verb refers not to an occurrence, but to the result of a task performance. Ryle (1978, p143-4) writes: “one big difference between the logical force of a task verb and the corresponding achievement verb is that in applying an achievement verb, we are asserting that some state of affairs obtains over and above that which consists in the performance, if any, of the subservient task activity.” Due to the logical independence of task verbs and achievement verbs, a task verb can be used to describe actions and performances, without presupposing any corresponding achievement verbs, and vice versa. Ryle believes that many verbs used to describe the intellectual lives of human beings are actually achievement verbs without any corresponding task verbs. Now we ask: Is “think” also an achievement verb without any corresponding task verb?

Ryle (1971, p292) clearly recognises that the verb “think” can refer to both beliefs and opinions. In *CM*, “believe” is regarded as a dispositional verb that refers to a tendency manifested through diverse actions which I perform from time to time, not only theoretical exercises of stating propositions, but also making some executive and imaginative moves and having certain feelings. So, if I think or believe that Ryle’s style is informal, I would not only be citing passages which were written by Ryle in a conversational vein and pointing out stylistic differences between standard academic prose and Ryle’s writings and so on. This tendency is an achievement in the sense that it is not an occurrence, but a state at which one arrives. Hence, the verb “think” does behave like an achievement verb: especially when used with a “that” clause, it expresses a thought. A thought, we must notice, is not something that goes on within the thinker; it is portrayed as a static object with which the thinker enters into a relation of ownership or possession. If I say that Mozart “thought” of a new composition on a certain occasion, I will be saying that Mozart was then *prepared* to express that new composition in some way, e.g. by playing it or noting it down. A thought, therefore, is a *state of being prepared for a final performance*, through which the thought is manifested and from which it derives its content. If Mozart were not prepared to *perform* his composition, I could rightfully doubt whether he had such thoughts at all and also whether his thoughts, if there were any, were *about* the composition.

Ryle (1978, p272) discusses this issue in terms of theory and theorising, where theories are “products of pondering” that the thinking subject comes to possess. He writes,

*“…in talking of building theories I am not referring only to the classical examples of famous discoveries but to a class of tasks in which all people who have had any education participate in some degree on some occasions. The housewife trying to find out whether a carpet will fit a floor is engaged in an unambitious task of theorising. She is investigating something and the results of her investigations will be statable. Both what she reports to her husband and what she does with the carpet will show what theory she has reached, since her morning’s work with tape-measure, pencil and paper was preparing her both to lay the carpet this way round and not that, and to tell her husband that the carpet will go there that way round, since the shape and size of the floor and of the carpet are so and so. I am also using the word ‘theory’ to cover the results of any kind of systematic inquiry, whether or not these results make up a deductive system. An historian’s account of the course of a battle is his theory”*.

Therefore, it seems, to possess or master a theory is to acquire the ability or the competence to state the theory or otherwise apply it. If I have the theory about a drug to cure cancer, it will merely not suffice to state in it words, write a paper on it, or to lecture about it to scientists. Such a theory is futile unless it is applied, unless the drug is manufactured and tested.

Now, if *having a thought* is an achievement, is it preceded by a task performance? For example, under an information-processing model of thinking, thinking is nothing over and above a sequence of configurations of the brain or the states of a computational mind arising from those configurations. Here, thinking, as a *process* that consists solely of achievements, is not a distinct task performance. Ryle (1978, p 286) dismissively compares this interpretation of thinking to “describing a journey as constituted by arrivals, searching as constituted by findings, studying as constituted by examination triumphs, or, in a word, trying as constituted by successes”.

Ryle (1971, p470) says that thoughts are what the *act of thinking* “incorporates or terminates in, if it prospers”. In *CM*, Ryle (1978, p268-269) invokes this distinction between thinking and thoughts:

*“We must distinguish between the sense in which we say that someone is engaged in thinking something out from the sense in which we say so and so is what he thinks, i.e., between the sense of ‘thought’ (here synonymous with the process of thinking) in which thought can be hard, protracted, interrupted, careless, successful or unavailing from the sense in which a person’s thoughts are true, false, valid, fallacious, abstract, rejected, shared, published, or unpublished. In the former sense we are talking about the work in which a person is at times and for periods engaged. In the latter sense we are talking about the results of such work”*.

If a thought is really a state of preparedness, then *thinking of thoughts*, as the act that underlies it, must be a preparation of some sort. In the first place, we must realise that “thinking” denotes several disparate activities. It is not a homogeneous activity, and is distinct from all other observable activities which we perform when we are reflecting, meditating or pondering on some topic. Secondly, thinking cannot be reduced to these concrete activities themselves; it forms a *programme* of which these activities are parts. Thus, a “thick” description of thinking must portray it as an act of *labouring towards a particular goal*. It must emphasise the very manner in which thinking is conducted: the tentativeness, the caution, the experimental temper, the spirit of rehearsal inherent in the concrete activities that constitute thinking. The *preparedness*, which embodies the possession of thoughts and which is manifested when thoughts are articulated or applied, is absent from the concrete activities that constitute thinking. On the contrary, one performs those activities with *an intention to prepare oneself* for a final performance. Thus, acts of thinking are *parasitic* on a final performance. This is what Ryle (1971, p478-479) calls *intention-parasitism* of thinking.

## Narratives of Thinking

How do we produce narratives of thinking? According to Ryle (1951, p74ff), when asked for such a narrative, we *assume* that our pensive labours have been parasitic on a final performance. This assumption of intention-parasitism makes significant reconstruction of thinking possible, facilitating the description of content which forms the “tenor of thinking” (Ryle 1971, p404) and which alone tells us how thinking culminated in a final achievement. Hence, the thinker must re-evaluate the success/failure tests he previously undertook in the light of the achieved results. He must reconstruct his thinking within a “bottom-up” model of intention-parasitism: he focusses his description on the results of the sub-ponderings, which cumulatively lead to the ultimate result of pondering. Now, it is only this re-evaluation – which is nothing more than a *tactical history* of thinking – that the thinker presents when he is asked to produce an account of his thinking, because intention-parasitism is the only retrospectible feature of thinking which preserves its content and significance. If, instead of a *tactical history* of thinking, we present only a *chronicle* of images, sounds and other expressions that occurred to us while thinking, we fail to capture those characteristics of thinking which are rooted in its essential intention-parasitism.

However, even a tactical history of thinking reconstructs thinking from its available results and, therefore, cannot record the acts of thinking itself. Ryle (1978, p272) shows that reporting a theory is just a path-showing or a path-using move, whereby one points out or uses a path which has already been made, whereas theorising is a path-making move. To describe such a move is by no means just an exercise of pointing out or using the path which is created by that very same path-making move. Hence, an accurate report of theory building takes into account the actual mental efforts which are put into the production of a theory. The point being made here is that reporting thoughts is not the same thing as reporting the process of thinking that led to them. Reports of products of pondering do not capture what the labours of thinking are like. Since we cannot retrospectively monitor our acts of thinking, our account of thinking remains inaccurate. At least some aspects of thinking always remain private.

Yet, it must be borne in mind that Ryle believes that it is through intention-parasitism that we can somewhat grasp what the labours of a thinker were like. It seems important to distinguish Ryle’s position from that of Dennett (1998, p212-213) who interprets every “introspective” venture as an attempt to build a story about oneself, which, as a product of cultural conditioning, cannot capture the actual nature of cognitive processes. On the contrary, Ryle believes that narratives of thinking, conditioned by the assumption of intention-parasitism, capture the *actual* content of thinking. This, I believe, has an important consequence in the context of a recent debate regarding Dennett’s philosophy. Ross and colleagues (Ross *et al*., 2000, p6) seem to imply that for both Ryle and Dennett, what is available to a cognitive agent is “merely processed information, and judgments about the source and interpretation, in natural language, of that information”. Hence, the ascription of content to cognitive processes on the basis of that processed information cannot strictly be called veridical or non-veridical, since no criterion of veridicality is possible at all. But I argue that this is not what Ryle would like to say. For him, the content ascribed to cognitive processes does constitute at least one actual aspect of those processes. In this respect, thinking always remains describable, howsoever inaccurately, and is never completely private.

However, thinking cannot be described within this framework of intention-parasitism, in cases of *non-achieving thinking*, where acts of thinking do not quite terminate in thoughts. For example, “…a poet, essayist or philosopher may be trying hard to find the word, phrase or argument that he needs, but the time when he is thinking what to say is the time when he still has nothing to say” (Ryle 1951, p69). We cannot decide how to portray such acts of thinking as parasitic on some actual goal, thereby failing to produce a meaningful narrative of thinking in such cases. For Ryle (1978, p269), an accurate account of thinking would be possible in such cases only if thinking consisted of “recordable operations actually executable by particular people at particular stages of their ponderings”. The impossibility of such an account in these instances shows that acts of thinking are generally indescribable *just as they happen*, outside the framework of intention-parasitism. We can only try to compensate indescribability by constructing narratives around results of thinking rather than thinking itself.

To my mind, this has something to do with what Ryle (1971, p474) calls the *circumstance-detachment* of thinking. For example, though one may think out a solution to a mathematical problem with pen and paper, the act of thinking does not strictly depend on pen and paper. But a first-order activity like writing a solution of a problem depends on external circumstances. This circumstance dependence makes, in turn, a genealogical reconstruction of first-order activities possible. In the case of thinking, we have only the products of the act after the act is over, simply because thinking may not be conditioned by external circumstances at all. Therefore, thinking cannot be described *just as it happens, just as it arises from its conditions*. Under this aspect, it remains private.

## Concluding Remarks [See also Figure 1]

**Figure 1:**
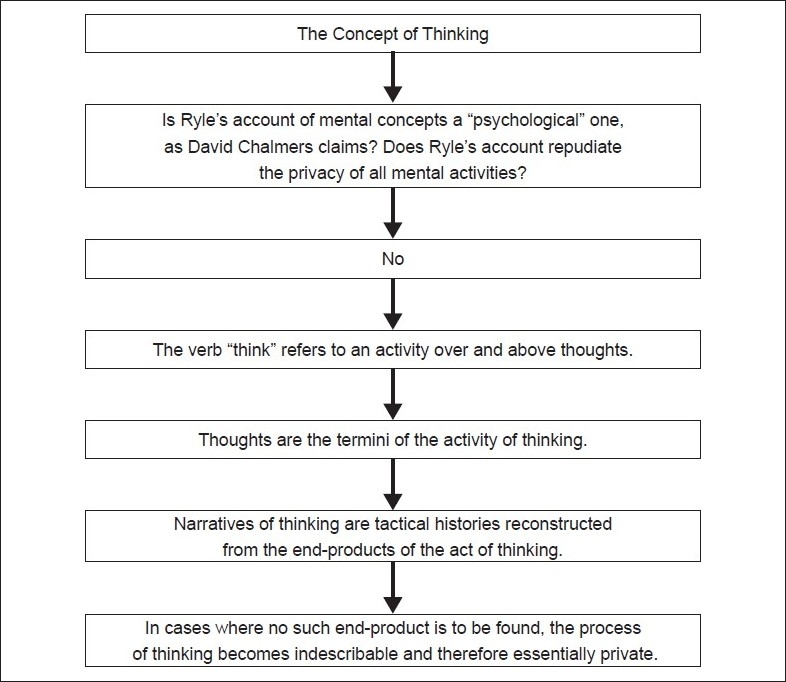
Flowchart of paper

This, however, does not mean that the conception of thinking, as an activity conditioned just by a thinker’s efforts, is a useful fiction. For Ryle, the word “think” does refer to circumstance-independent activities, which, though accessible to the thinker, are indescribable afterwards. This conception of thinking, which originates in *CM* itself, stands in clear opposition with the “psychological” interpretation of mental concepts ordinarily ascribed to Ryle, for it does not explain thinking in terms of observable behaviour or behavioural dispositions. This is because it understands thinking as an autonomous, circumstance-independent sequence of activities and places it outside the framework of intention-parasitism. Thus, it severs the ties of thinking with the describable world and emphasises its privacy.

## Take home message

In sum, we must not exaggerate Ryle’s attack on privacy of mental acts, given that it deals with only one aspect of mental acts. Therefore, ordinary “psychological” interpretation of Ryle’s work, which adopts polemic as one of its central premises, cannot be accepted entirely.

## Questions That This Paper Raises

1. Does the verb “think”, for Ryle, refer to activities over and above thoughts themselves?
2. What, according to Ryle, is the relation between thoughts and the thinking of thoughts?
3. How, according to Ryle, can a narrative of thinking be prepared?
4. Is there any instance of thinking which is completely indescribable?
5. How can such indescribability be reconciled with the attack on privacy that Ryle launches in *The Concept of Mind*?

## About the Author

*Nilanjan Das is pursuing a postgraduate degree at the Department of Philosophy, Jadavpur University, West Bengal, India. His chief interests lie in logic, epistemology and the philosophy of mind. He has also been traditionally trained in Nyaya and Vedanta.*
